# Progressive respiratory failure in a term neonate with ABCA3 surfactant deficiency: Beyond the common causes of respiratory distress

**DOI:** 10.1177/19345798251371042

**Published:** 2025-08-25

**Authors:** Andrew M. Beverstock, Hillary C. Lee, David S. Moreno McNeill, Morcos Hanna

**Affiliations:** 1 Section of Neonatology, Department of Pediatrics, Baylor College of Medicine/Texas Children’s Hospital, Houston, TX, USA; 2 Section of Pediatric Pulmonology and Lung Transplant, Department of Pediatrics, Baylor College of Medicine/Texas Children’s Hospital, Houston, TX, USA

**Keywords:** genetic lung disease, respiratory distress, surfactant deficiency

## Abstract

**Background:**

Most cases of respiratory distress in term neonates are due to transient tachypnea of the newborn (TTN), respiratory distress syndrome (RDS), or air leak syndromes. Genetic surfactant deficiencies are rare causes of respiratory distress. Among these, mutations in the *ABCA3* gene disrupt surfactant metabolism and can lead to severe, treatment-refractory respiratory failure. While commonly considered in preterm infants, surfactant dysfunction should also be considered in term infants with unexplained and persistent hypoxemia.

**Case:**

We present a case of a 38-weeks term female infant with fetal growth restriction who developed respiratory distress shortly after birth. She initially responded to continuous positive airway pressure (CPAP) and surfactant but required escalating respiratory support and multiple re-doses of surfactant. Standard infectious and cardiopulmonary evaluations were unrevealing. Given her persistent oxygen requirement and small-for-gestational-age status, genetic testing was pursued. Whole genome sequencing identified bi-allelic pathogenic variants in the *ABCA3* gene, consistent with pulmonary surfactant metabolism dysfunction type 3. Despite six doses of surfactant, antibiotics, and inhaled nitric oxide, the patient’s respiratory status deteriorated. Lung transplantation was not feasible due to size and clinical condition. The family elected to transition to comfort care.

**Conclusion:**

This case highlights the importance of considering genetic surfactant disorders, including ABCA3 mutations, in term neonates with refractory respiratory distress. Early genetic testing can guide management and avoid potentially harmful or ineffective interventions. While some therapies offer transient improvement, outcomes remain poor, and definitive treatment via lung transplantation is limited by size and disease progression. Future research should focus on gene-specific therapies and earlier diagnosis.

## Case presentation

**Figure 1.**
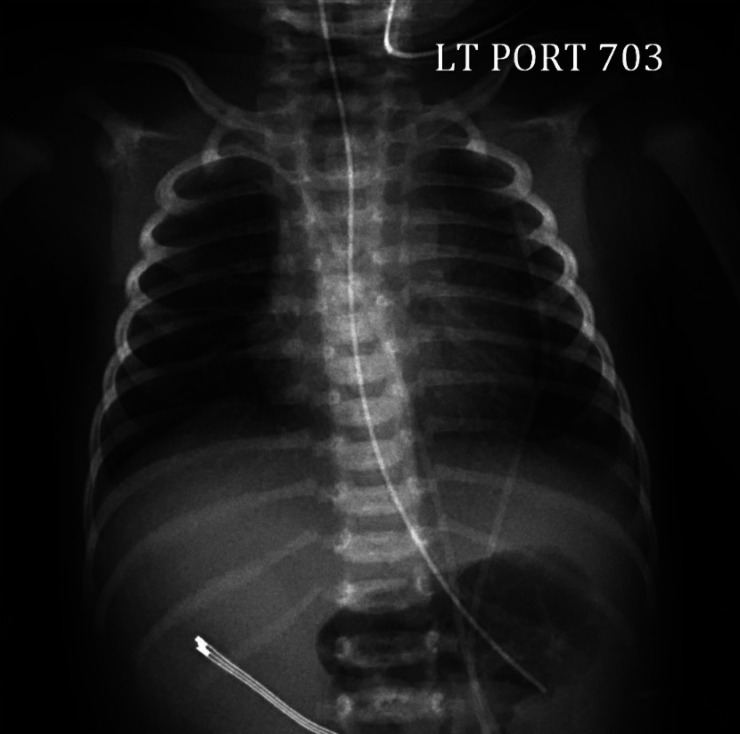
Chest X-ray of infant obtained at 90 min of life on CPAP.

A female was born at 38-weeks via spontaneous vaginal delivery to a G3P2 mother. Her prenatal course was notable for fetal growth restriction (3^rd^ percentile). Maternal prenatal labs and placental pathology were unremarkable. She initially cried but then became apneic at 1 min of life, requiring 2 min of positive pressure ventilation (PPV) followed by continuous positive airway pressure (CPAP). Her Apgar scores were 5, 6, and 9 at 1, 5, and 10 min, respectively. She was transferred to the neonatal intensive care unit on CPAP 6 where her fraction of inspired oxygen (FiO_2_) was weaned to 35%. Her initial chest X-ray is shown in Figure 1. Her initial physical exam was unremarkable, except for being symmetrically small for gestational age. Her birth weight was 2.23 kg (5^th^ percentile), and head circumference was 31 cm (6^th^ percentile). Her vitals were normal except for comfortable tachypnea. She had clear lungs to auscultation and no evidence of labored breathing.

Due to her persistently high FiO_2_ requirement (30%–40%), surfactant was administered via less invasive surfactant administration (LISA) at 14 h of life. Her FiO_2_ requirement gradually improved over the next 12 h. However, within the following 48 h, her oxygen requirement increased back to 50%, leading to the administration of a second dose of surfactant via LISA. Her FiO_2_ initially improved again but then rose to 30% after 24 h. Blood cultures obtained after birth were negative, and empiric antibiotics were discontinued at 48 h of life.

The infant remained on CPAP with a progressively rising FiO2 requirement. An echocardiogram on day of life 4 revealed evidence of persistent pulmonary hypertension of the newborn (PPHN). By day 7, she developed severe respiratory distress and was intubated, requiring 100% oxygen, inhaled nitric oxide, sedation, and neuromuscular blockade to maintain an oxygen saturation (SpO_2_) above 90%. A repeat sepsis evaluation was negative, but *E. Coli* was detected in a tracheal aspirate, leading to 14-days treatment course of antibiotics. A repeat echocardiogram showed resolution of PPHN. However, despite appropriate treatment, she continued to require high ventilator settings and a high FiO_2_. Given her unexplained small-for-gestational-age status and persistently high oxygen requirement, the genetics team was consulted and recommended a whole genome sequence.

## Differential diagnosis

The primary differential diagnoses for a term infant with respiratory distress are transient tachypnea of the newborn (TTN), respiratory distress syndrome (RDS), air leak syndromes like pneumothorax, persistent pulmonary hypertension of the newborn (PPHN), respiratory and systemic infections, and childhood interstitial lung diseases (chILD).

In this case, TTN and RDS were excluded based on the progressive nature of the condition. TTN is generally benign and self-limiting, which improves with supportive care.^1^ This patient’s respiratory distress worsened over time, which is not consistent with TTN. Similarly, RDS peaks within the first 24–48 h, but typically improves over the first 72 h with appropriate interventions such as respiratory support and exogenous surfactant administration. This infant was initially thought to have RDS, and her early improvement after surfactant administration supported that diagnosis; however, the recurrence and worsening of symptoms beyond the typical course of RDS, along with a reduced response to additional surfactant doses, raised concern for an alternative underlying process. Air leak syndrome was excluded on chest radiography, which is highly sensitive and specific for pneumothorax. If there is continued clinical suspicion, lung ultrasound may be used to detect smaller air leaks that may not be visible on radiography.^2^ Chest radiograph also made congenital diaphragmatic hernia (CDH) and other congenital lung malformations less likely. Systemic infection was excluded based on negative blood cultures. Respiratory tract infection was considered in this infant due to the positive tracheal aspirate for *E. coli,* but there was no improvement with antibiotic administration, making this an unlikely primary cause of her respiratory insufficiency. Echocardiogram ruled out congenital heart disease and initially showed evidence of PPHN, which was appropriately treated. However, the infant continued to have significant respiratory distress and hypoxemia despite a subsequent echocardiogram showing resolution of PPHN.

## Diagnosis

Rapid Trio whole genome sequencing (WGS) was sent on day of life 11. It resulted on day of life 24, revealing two autosomal recessive variants in the ATP-binding cassette transporter A3 (*ABCA3*) gene with one variant inherited from each parent. Both parents were heterozygous for different pathogenic sequence variants in the *ABCA3* gene, which are known to cause pulmonary surfactant metabolism type 3, resulting in homozygous disease for this infant.

## Clinical course

**Figure 2.**
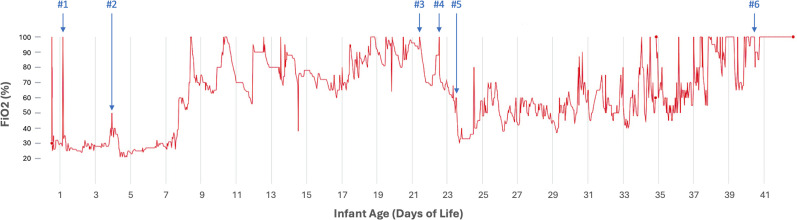
Graph depicting FiO_2_ requirement in red. Blue arrows mark surfactant administration to infant via LISA (#1-#2) and endotracheal tube (#3-#6).

The lung transplant team was consulted given the diagnosis, but she was not eligible for lung transplantation at the time due to her small size. She received three additional daily doses of surfactant, but these provided only temporary benefits (Figure 2). Treatment with azithromycin and hydroxychloroquine was initiated, along with a 3-days course of pulsed methylprednisolone. However, this also resulted in only short-term improvement, and her ventilator support was unable to be weaned. A 6^th^ dose of surfactant was administered towards the end when she was extremely ill with minimal effect. The family chose to redirect care to allow for a natural death.

A full autopsy was performed, which was also consistent with ABCA3 surfactant deficiency. Her lungs were twice the expected weight for her size (patient’s lung size 128g compared to 60g expected) with an elevated lung to body weight ratio of 0.049 (expected 0.018-0.02). Additionally, histopathology revealed evidence of interstitial lung disease with patchy areas of desquamative interstitial pneumonia and pulmonary alveolar proteinosis as well as diffuse alveolar septal thickening with type 2 pneumocyte hyperplasia and minimal interstitial fibrosis. There was also evidence of long-standing systemic hypoxia with extramedullary hematopoiesis in the lungs, spleen, and liver, as well as periportal and centrilobular hepatocellular iron deposition. These postmortem findings are all consistent with a diagnosis of ABCA3 surfactant deficiency.^3^

## Discussion

Pulmonary causes of respiratory failure in term neonates include TTN, RDS due to surfactant deficiency or dysfunction, meconium aspiration syndrome, pneumonia, and air leak syndromes.^4,5^ Less common pulmonary conditions should also be considered, including CDH and structural abnormalities of the lung or airway that may have not been identified prenatally.^6^ Beyond pulmonary etiologies, respiratory distress can result from conditions affecting other systems, including infections, cardiac anomalies, airway obstruction, metabolic disturbances, neurologic injury, and hematologic disorders.^7,8^ Genetic syndromes, particularly those affecting surfactant production or alveolar development, though rare, should also be considered in infants with persistent or unexplained respiratory failure.

The most common mutations responsible for these disorders are found in surfactant protein B, surfactant protein C, and in ABCA3.^9^ Of these, ABCA3 surfactant protein disorder is the most common with an incidence of approximately 100 live-born infants affected in the United States every year.^10^ *ABCA3* gene encodes a transport protein essential for inserting phospholipids into the surfactant complex within type II pneumocytes, a crucial process for maintaining proper surfactant function and reducing surface tension in the alveoli.^11,12^ Mutations in *ABCA3* disrupt surfactant production with variable clinical presentation. Pathogenic ABCA3 mutation phenotypes range from lethal respiratory distress in newborns to chronic coughs that appear later in childhood, depending on the specific mutation and level of protein expression.^13^ Infants with bi-allelic mutations have a 5-years survival of under 20%.^14^

Our case highlights the importance of genetic testing, which can be used to make this diagnosis without exposing infants to the risks of invasive lung biopsy or bronchoscopy. ABCA3 surfactant deficiency can be diagnosed through genetic testing. For our patient, a Rapid Trio WGS was ordered, though genetic surfactant metabolism panels might provide results more quickly and at lower cost. In addition, WGS may reveal additional genetic information with important future implications for a patient that were not relevant during their initial hospitalization, raising important ethical and privacy considerations.^15^

Infants with genetic surfactant deficiencies may initially respond to intratracheal surfactant administration with some improvement, but this response is often short-lived. There is also concern that repeated doses of surfactant may increase pulmonary inflammation, potentially worsening the condition over time. We noted a blunted response with repeated doses, which should raise suspicion for atypical causes of respiratory distress, such as infection or surfactant deficiencies. When comparing our case to other recently published literature, our infant displayed a more severe phenotype with a poorer response to surfactant administration.^16^ The only curative therapy for genetic surfactant deficiencies is bilateral lung transplantation, which carries high morbidity and mortality risks.^17^

Due to the rarity of these conditions, there have been few randomized clinical trials regarding additional therapies such as hydroxychloroquine, azithromycin, corticosteroids, and cyclosporine. Some case reports suggest that hydroxychloroquine may be beneficial due to its immunomodulatory effects,^18^ but a recent phase 2 randomized controlled trial found limited efficacy in patients with genetic surfactant deficiencies.^19^ Research indicates that hydroxychloroquine’s effectiveness varies depending on the specific ABCA3 variant, highlighting the need for individualized treatment based on genetic testing.^20^ Similarly, azithromycin has been successfully used in some patients, although the evidence supporting its effectiveness is primarily based on case reports.^21^ Additionally, corticosteroids can also be effective. In animal models, systemic dexamethasone upregulates ABCA3 expression,^22^ and systemic steroids have shown benefits in some cases.^23^ However, corticosteroids can impair growth,^24^ which is a significant concern for neonates, as growth is crucial for becoming eligible for lung transplantation. Cyclosporine has been proposed as a potential therapy but has not been extensively studied.^25^ Gene therapy might offer future hope since ABCA3 deficiency is a monoallelic condition, but this option is not yet available.^26^

Our case highlights the possibility of early diagnosis for this disorder. When term infants with respiratory distress do not respond appropriately to usual treatment methods, consider early genetic testing or lung biopsy to look for rare causes, including genetic surfactant deficiencies.

## Lessons for the clinician

• Inherited surfactant deficiency should be considered as a diagnosis in any term infant with respiratory distress who shows a blunted response to surfactant administration.
• Consider sending genetic testing for surfactant protein deficiency if the disorder is suspected since these infants are at risk of acute decompensation to the point of requiring extracorporeal life support.
• There may be a role for systemic corticosteroids, hydroxychloroquine, and azithromycin, but each patient’s response must be carefully assessed.
• The only curative treatment for pulmonary surfactant metabolism dysfunction type 3 is lung transplantation. Early referral to a lung transplantation center is indicated.
